# Optimal Dose and Method of Administration of Intravenous Insulin in the Management of Emergency Hyperkalemia: A Systematic Review

**DOI:** 10.1371/journal.pone.0154963

**Published:** 2016-05-05

**Authors:** Ziv Harel, Kamel S. Kamel

**Affiliations:** 1 Division of Nephrology, St Michael’s Hospital, University of Toronto, Toronto, Canada, 2 Department of Medicine, St Michael’s Hospital, University of Toronto, Toronto, Canada; 3 Keenan Research Centre, Li Ka Shing Knowledge Institute of St Michael’s Hospital, University of Toronto, Toronto, Canada; Sao Paulo State University, BRAZIL

## Abstract

**Background and Objectives:**

Hyperkalemia is a common electrolyte disorder that can result in fatal cardiac arrhythmias. Despite the importance of insulin as a lifesaving intervention in the treatment of hyperkalemia in an emergency setting, there is no consensus on the dose or the method (bolus or infusion) of its administration. Our aim was to review data in the literature to determine the optimal dose and route of administration of insulin in the management of emergency hyperkalemia.

**Design, Setting, Participants, & Measurements:**

We searched several databases from their date of inception through February 2015 for eligible articles published in any language. We included any study that reported on the use of insulin in the management of hyperkalemia.

**Results:**

We identified eleven studies. In seven studies, 10 units of regular insulin was administered (bolus in five studies, infusion in two studies), in one study 12 units of regular insulin was infused over 30 minutes, and in three studies 20 units of regular insulin was infused over 60 minutes. The majority of included studies were biased. There was no statistically significant difference in mean decrease in serum potassium (K^+^) concentration at 60 minutes between studies in which insulin was administered as an infusion of 20 units over 60 minutes and studies in which 10 units of insulin was administered as a bolus (0.79±0.25 mmol/L versus 0.78±0.25 mmol/L, *P* = 0.98) or studies in which 10 units of insulin was administered as an infusion (0.79±0.25 mmol/L versus 0.39±0.09 mmol/L, *P* = 0.1). Almost one fifth of the study population experienced an episode of hypoglycemia.

**Conclusion:**

The limited data available in the literature shows no statistically significant difference between the different regimens of insulin used to acutely lower serum K^+^ concentration. Accordingly, 10 units of short acting insulin given intravenously may be used in cases of hyperkalemia. Alternatively, 20 units of short acting insulin may be given as a continuous intravenous infusion over 60 minutes in patients with severe hyperkalemia (i.e., serum K^+^ concentration > 6.5 mmol/L) and those with marked EKG changes related to hyperkalemia (e.g., prolonged PR interval, wide QRS complex) as an alternative to 10 units of short acting insulin. Because the risk of hypoglycemia is increased with using large insulin doses, sufficient glucose (60 grams with the administration of 20 units of insulin and 50 grams with the administration of 10 units) should be given to prevent hypoglycemia, and plasma glucose should be frequently monitored.

## Background

Hyperkalemia is a common electrolyte disorder that can result in fatal cardiac arrhythmias.[1] Successful management of acute hyperkalemia involves protecting the heart from arrhythmias with the administration of calcium, shifting potassium (K^+^) into the cells, and enhancing the elimination of K^+^ from the body. Intravenous short acting insulin has been recommended as the first-line agent used for shifting K^+^ into cells in treatment of hyperkalemia in an emergency setting.[2–4] The recommended dose reported in a number of textbooks of Internal Medicine and of Nephrology is 10 units of regular insulin administered as an intravenous bolus; however, the evidence supporting such a recommendation is not clear.[5–8] (Table 1) Although prior systematic reviews have attempted to define the optimal dose and method (bolus or infusion) of administration of insulin of insulin in the management of acute hyperkalemia, these have only focused on a small number of randomized controlled trials or did not include a quality assessment, which limit the generalizability of the results.[4, 9, 10] Therefore, our aim was to review data in the literature from both randomized controlled trials and observational studies to determine the optimal dose and route of administration of insulin in the management of emergency hyperkalemia.

**Table 1:** Recommended regimens for administration of insulin in the treatment of acute hyperkalemia.

Reference	Regimen
Brenner and Rector’s the Kidney	10 units intravenous bolus of regular insulin with 50 ml of D50W (25 grams of glucose)
Harrison’s Principles of Internal Medicine	10 units intravenous bolus of regular insulin with 50 ml of D50W (25 grams of glucose)
The Washington Manual of Medical Therapeutics	10–20 units intravenous bolus of regular insulin with 50–100 ml of D50W (25–50 grams of glucose)
UpToDate	10 units intravenous bolus of regular insulin with 50 ml of D50W (25 grams of glucose) **OR** 10 units of regular insulin with 500 ml of D10W (50 grams glucose) over 60 minutes)

## Methods

We performed this systematic review in accordance with the preferred reporting items for systematic reviews and meta-analyses guidelines.[11] (S1 Appendix)

### Study Selection and outcomes

We considered articles to be eligible for inclusion if they reported on using insulin in the management of hyperkalemia. We included all study designs that used a standardized protocol except for case reports. We also excluded articles from further review if they fulfilled one or more of the following criteria: patients less than 18 years of age; hyperkalemia that occurred in an operative setting; and articles in which there were co-interventions for the management hyperkalemia (e.g. concurrent use of diuretics, resins etc.).

Our primary outcome was the mean change in serum K^+^ concentration at 60 minutes after starting the administration of insulin.

### Literature Sources

We searched three electronic databases: OVID Medline (1946 to March 9, 2015), EMBASE (1946 to 2015 Week 10) and the Cochrane Central Register of Controlled Trials (CENTRAL) (1993 to February 2015) using a search strategy developed with an experienced health informatics specialist (S2 Appendix). We applied no language restrictions, and reviewed the bibliographies of identified articles to locate further eligible studies. In addition, we searched abstracts of the last five years from the annual meeting of the American Society of Nephrology for relevant articles. Where necessary we contacted corresponding authors for additional missing data; however, in one study we had to estimate serum K^+^ concentration values from the graphs provided.[12]

### Data abstraction

The two authors (ZH and KK) scanned titles and abstracts for initial selection. Selected articles were reviewed in full and were independently assessed for eligibility by the two authors. Discrepancies were resolved by consensus.

We abstracted the following data from each included article: study design; population; the route and dose of insulin administered; mean initial serum K^+^ concentration and mean serum K^+^ concentration at 60 minutes; serum insulin concentrations at various time points; the total amount of glucose infused; and the occurrence of hypoglycemia.

### Quality Assessment

Two reviewers (ZH and KK) independently assessed the risk of bias in the included studies without blinding to authorship or journal by using the predefined checklists of the Cochrane Database of Systematic Reviews.[13, 14] The included studies consisted of randomized and non-randomized trials, and we used the appropriate Cochrane quality assessment checklists specific to the study design.[13, 14] For non-randomized trials, the overall risk of bias for each study was summarized as low, moderate, serious or critical, based on the Cochrane checklist criteria.[13] Similarly, for randomized trials, we also carried out an overall assessment of the risk of bias (low, high or unclear) based on the responses for the selected criteria for the Cochrane checklist.[14] Discrepancies between the two reviewers were resolved by consensus.

### Statistical Analysis

We were aware that studies published in this area were highly heterogeneous and thus no meta-analysis was planned. We used descriptive statistics to compare differences in the mean decrease in serum K^+^ concentration at 60 minutes between patients receiving 10 units of insulin as an intravenous bolus, patients receiving 10 units of insulin as an intravenous infusion, and patients receiving 20 units of insulin as a continuous infusion. Continuous variables were expressed as a mean (± standard error) and compared using an unpaired t-test. Where indicated, we converted standard deviations to standard errors to aid in comparison among the studies. We considered a *P*-value less than 0.05 to be statistically significant. All statistical tests were conducted using SAS 9.2 (SAS Institute, Cary, NC).

## Results

**Fig 1:**
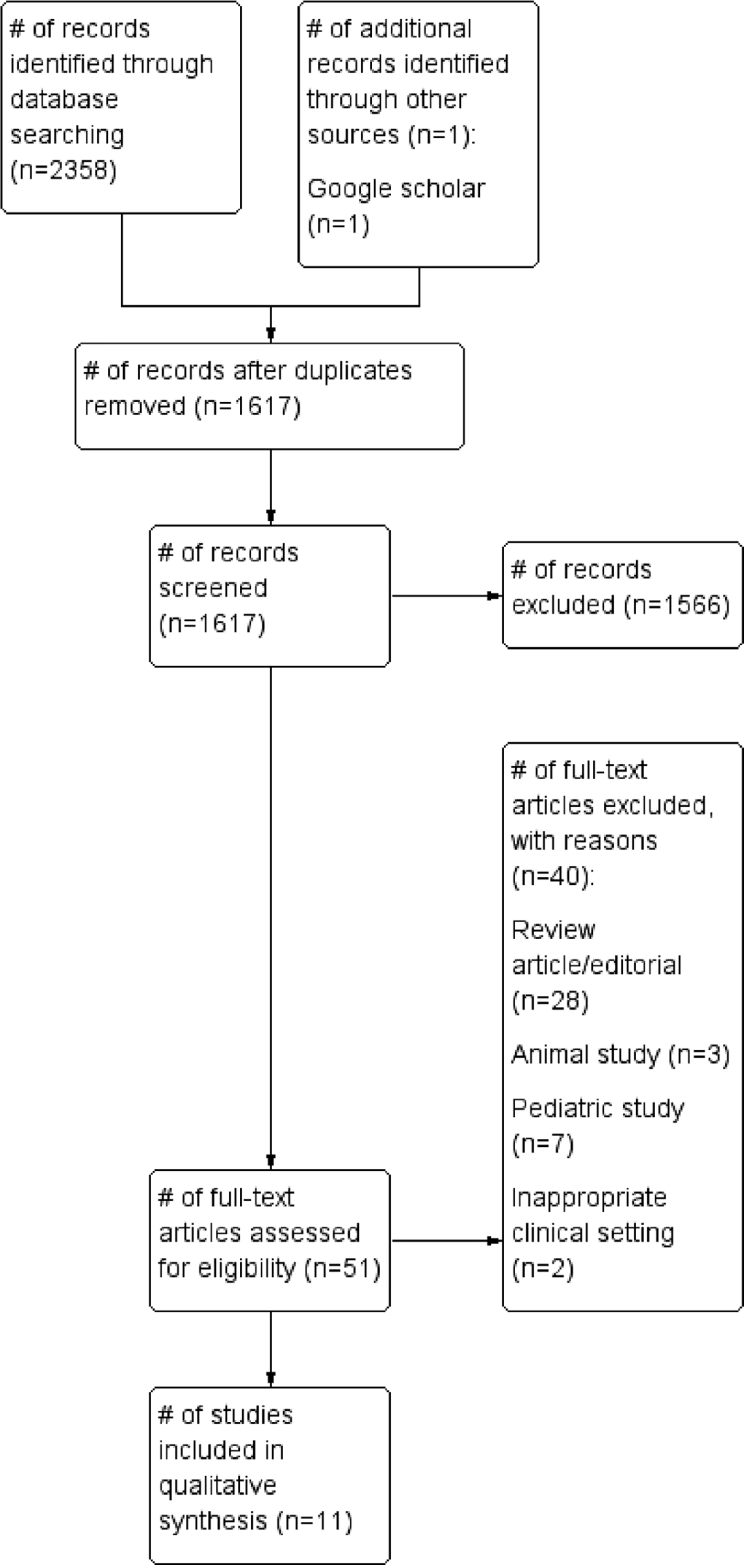
Study selection.

We identified 51 relevant articles from our literature search. After applying our exclusion criteria, eleven studies were included in the review.[12, 15–23] Of these, three were prospective non-randomized crossover trials, three were prospective non-randomized trials, two were prospective randomized crossover trials, two were prospective randomized trials, and one was a case series. (Fig 1 and Table 2). None of the trials used a control group (no intervention) as a comparator.

**Table 2:** Included studies.

Study^£^	Design	Population	Insulin dose	Insulin delivery method (Intravenous)	Mean initial potassium concentration(mmol/L±SE)	Mean decrease in potassium concentration at 60 minutes (mmol/L ±SE)	Mean repeat potassium concentration(mmol/L±SE)	Plasma insulin concentration (μU/ml± SE)	Glucose dose (grams)	Hypoglycemic episodes N (%)
Allon et al.[12] (n = 12)	Prospective,non-randomized crossover study	Outpatients on chronic hemodialysis (in the United States)	10 units	Bolus	5.48 ±0.21	0.53±0.25*	0 min: 5.48 ±0.2130 min: 4.88 ±0.31^¥^^α^45 min: 4.83±NR60 min: 4.88 ±0.31^¥^^α^	0 min: 8.8±2.215 min:319 ±3930 min: 185±3345 min:98.8± 26.360 min: 61.9±21.6	25	9 (75)
Chothia et al. [24](n = 10)	Prospective, randomized, cross-over study	Non diabetic patients on chronic hemodialysis patients (in South Africa)	10 units	Bolus	6.01±0.28^¥^	0.83±0.17^¥^	0 min: 6.01±0.28^¥^60 min:5.18±0.24^¥^	0 min: 28.4±10.3^¥^10 min:547.5 ± 110^¥^20 min:467.4 ± 68.6^¥^40 min: 200.6 ± 45.9^¥^60 min: 92.3±20.9^¥^	50	2 (20)
Lens et al.[22](n = 10)	Prospective,non-randomizedstudy	Hospitalizedpatients withAKI or CKD (in Spain)	10 units	Bolus	6.7 ±0.2^¥^	1.0±0.1^¥^	0min: 6.7 ±0.06^¥^30 min: 5.8 ±0.06^¥^60 min: 5.7 ±0.06^¥^180 min: 6.1±0.09^¥^ 360 min: 6.4 ±0.09^¥^	NR	40	2 (20)
Ljutic et al.[23](n = 9)	Case series	Patients on maintenance hemodialysis (in Croatia)	10 units	Bolus	6.33±0.22	0.76±0.39	0min: 6.33±0.2230 min:5.59±0.2945min: 5.54±0.2860 min: 5.57±0.24	0 min: 14.77±0.8415 min: 267.44± 26.1330 min: 179.33±29.8745 min:123.16± 24.0160 min: 60.07±12.98	25	1 (10)
Mushtaq et al.[16](n = 5)	Prospective,non-randomizedstudy	Hospitalizedpatients withAKI or CKD(in Lahore,Pakistan)	10 units	Bolus	6.5±0.3	0.8±0.25	0min: 6.5±0.330 min: 5.9± 0.260 min: 5.7 ± 0.2180 min: 5.9±0.2360 min: 6.0 ± 0.2	NR	25	0
Ngugi et al.[19](n = 10)	Prospective,randomizedstudy	Hospitalizedpatients withAKI or CKD(in Nairobi,Kenya)	10 units	Infusion over 15 minutes	5.98±NR	1.14±NR	0min: 5.98±NR30 min: 5.13±NR60min:4.84±NR	NR	25	2 (20)
Duranay et al.[21](n = 20)	Prospective,non-randomizedstudy	Patients withCKD (in Turkey)	10 units	Infusion over 30 minutes	6.71±0.09^¥^	0.3±0.09^¥^	0min: 6.71±0.09^¥^30min: NR60 min: 6.41±0.11^¥^180 min: 5.72±0.13^¥^360 min: 5.97±0.12^¥^	NR	30	0
Mahajan et al.[17](n = 15)	Prospective,randomizedstudy	Patients withESRD (in India)	12 units	Infusion over 30 minutes	6.59±0.08^¥^	0.47±0.09^¥^	0min: 6.59±0.08^¥^30min: NR60 min: 6.12±0.11^¥^180min: 5.76±0.08^¥^360min:5.84±0.05^¥^	NR	25	1 (7)
Kim et al.[15] (n = 8)	Prospective,non-randomizedcross-overstudy	Outpatientson chronichemodialysis (in Korea)	5mU/kg/min(~20 units)	Infusion over 60 minutes	6.3±0.1	0.6±0.14	0min: 6.3±0.130 min: 6.0±0.160 min: 5.7±0.1	0 min: 9±1.560 min: 196±18	40	0
Allon et al.[25] (n = 8)	Prospective,randomizedcross-overstudy	Non-diabetic,outpatientson chronichemodialysis (in the United States)	5mU/kg/min(~20 units)	Infusion over 60 minutes	4.28±0.30	0.85±0.06	0 min:4.28±0.3030 min: 3.58 ± NR60 min: 3.43± NR	0 min: 12±215 min: 316±4930 min: 411±6145 min: 462±8360 min: 492±99	60	0
Blumberg et al.[20] (n = 10)	Prospective,non-randomizedcross-overstudy	Outpatientson chronichemodialysis (in the United States)	5mU/kg/min(~20 units)	Infusion over 60 minutes	5.62±0.33	0.92±0.40	0min:5.62 ±0.3330 min: NR60 min: 4.7±0.22	0 min: 14.56±3.3820 min: 281±18.6540 min: 332±20.8760 min: 354±21.79	25	5 (50)

*Abbreviations*: NR: Not reported; mmol/L: millimole per Litre; min: minutes; μU/mL: micro unit per Liter kg: kilogram; ESRD: end stage renal disease; CKD: chronic kidney disease; AKI: acute kidney injury, SE: standard error.

^£^n refers to the number of patients receiving insulin therapy.

*Estimated value from graph.

^¥^Standard deviations were converted to standard errors for comparison where appropriate.

^α^Values derived from chart in reference [4]

### Patient population

The eleven studies varied in their patient populations. Seven studies included outpatients on chronic hemodialysis[12, 15, 17, 18, 20, 23, 24], three included patients admitted to hospital with acute kidney injury (AKI) or chronic kidney disease (CKD)[16, 19, 22], and one included outpatients with CKD[21]. The definition of hyperkalemia differed among the included studies. Four studies[15–17, 21] defined hyperkalemia as a serum K^+^ concentration > 6 mmol/L, four studies[12, 19, 23, 24] defined it as a serum K^+^ concentration >5 mmol/L, and three studies[20, 22, 25] did not provide a definition for hyperkalemia. The number of patients included in each study varied from 5[16] to 20[21] (Table 2).

### Insulin dose, method of administration and glucose dose

In seven studies[12, 16, 19, 21–24], 10 units of regular insulin were administered. Of these studies, insulin was administered as a bolus in five studies[12, 16, 22–24], and as an infusion in two studies[19, 21] (one over 15 minutes and one over 30 minutes). One study[17] infused 12 units of regular insulin over 30 minutes, the data from this study were analyzed with the 10 units of insulin infusion group. Three studies[15, 20, 25] infused insulin in a dose of 5 iu per kg body weight per minute for 60 minutes (Table 2). This was extrapolated to the infusion of 20 units of insulin over 60 minutes based in a 70 kg subject.

Glucose was administered as 25 grams in 6 studies[12, 16, 17, 19, 20, 23], 30 grams in one study[21], 40 grams in two studies[15, 22], 50 grams in one study[24] and 60 grams (as an infusion over 60 minutes) in one study[25] (Table 2).

### Outcomes

It was not possible to perform a meta-analysis due to methodological and clinical heterogeneity among the included studies.

### Change in serum potassium and insulin concentrations

Table 2 shows the changes in the serum K^+^ concentrations over time for the included studies. In seven studies[15–17, 21–24] the mean initial serum K^+^ concentration was greater than 6 mmol/L; in three studies[12, 19, 20] it was between 5 and 6 mmol/L, and in one study[25], it was between 4 and 5 mmol/L.

The mean decrease in serum K^+^ concentration at 60 minutes ranged from 0.3±0.42 mmol/L to 1.14±NR (not reported) mmol/L. Among the three studies where insulin was administered as a 20 unit infusion over 60 minutes, the mean decrease in serum K^+^ at 60 minutes was 0.79±0.25 mmol/L. In the five studies, which administered 10 units of regular insulin, as a bolus, the mean decrease in serum potassium at 60 minutes was 0.78±0.25 mmol/L. There was no significant difference in the mean decrease in the serum K^+^ concentration when comparing the five studies, which administered 10 units of regular insulin, as a bolus, to the three studies in which 20 units of insulin was infused over 60 minutes (*P* = 0.98). Among the three studies where insulin was administered as an infusion of 10 or 12 units over 15–30 minutes, only two provided sufficient information to calculate the mean decrease in K^+^ concentration at 60 minutes, which was 0.39±0.09 mmol/L. There was no significant difference in the mean decrease in the serum K^+^ concentration when comparing the studies, which administered 10 units of regular insulin, as an infusion, to the three studies in which 20 units of insulin was infused over 60 minutes (*P* = 0.1)

Six studies reported plasma insulin concentrations. In the study by Allon et al.[12] where 10 units of insulin was administered as a bolus, insulin concentration peaked at 15 minutes (319±39 μU/mL) and subsequently declined. A similar trend was demonstrated in the study by Ljutic et al.[23], where insulin concentration peaked at fifteen minutes (267.44±26.13 μU/mL) and also subsequently declined. In the study by Chothia et al.[24], in which 10 units of insulin as a bolus and 50 grams of glucose were administered, the peak concentration of insulin occurred close to 10 minutes (547.5±110 μU/mL), and remained high during most of the study period. In contrast, among the 3 studies where 20 units of insulin was administered as an infusion, the plasma insulin concentration rose throughout the initial 60 minutes of the study. The mean plasma insulin concentration at 60 minutes was much lower in the study by Kim et al. (196±18 μU/ml)[15], compared to the measured levels in the study by Allon et al.[25] (492 ± 99 μU/ml), and in the study by Blumberg et al.[20] (354± 22 μU/ml), even though similar doses of insulin were administered.

### Hypoglycemia

Across all the studies reviewed, 22 patients (18%) experienced an episode of hypoglycemia. Studies where only 25 grams of glucose were administered had the highest incidence of hypoglyemia (30%). In study by Allon et al.[25], when 60 grams of glucose were given with the administration of 20 units of insulin, none of the patients developed hypoglycemia.

### Risk of bias

The majority of included studies were biased. Specifically, the risk of bias was serious for most of the non-randomized trials, and high for the randomized trials. (Tables 3 and 4)

**Table 3:** Overall risk of bias for non-randomized trials.

Study	Overall risk of bias
Allon et al.[12] (n = 12)	Serious
Lens et al.[22](n = 10)	Serious
Ljutic et al.[23] (n = 9)	Critical
Mushtaq et al.[16] (n = 5)	Critical
Duranay et al.[21] (n = 20)	Serious
Mahajan et al.[17] (n = 15)	Serious
Kim et al.[15] (n = 8)	Serious
Blumberg et al.[20] (n = 10)	Serious

**Table 4:** Overall risk of bias for randomized trials.

Study	Overall risk of bias
Chothia et al. [24] (n = 10)	Low
Ngugi et al.[19] (n = 10)	High
Allon et al.[25] (n = 8)	High

## Discussion

Hyperkalemia is a common electrolyte disorder that may lead to life threatening cardiac arrhythmias. Management of hyperkalemia involves protecting the heart from arrhythmias, shifting K^+^ into cells, and enhancing the elimination of K^+^. Insulin therapy is the most reliable and consistent method of shifting K^+^ into cells. K^+^ ions are kept inside cells due to the negative voltage in the cell interior. To shift K^+^ into cells, a more negative cell voltage is required. This is generated by increasing the flux through the sodium/potassium ATPase (Na-K ATPase) pump, as this is an electrogenic pump which exports three Na^+^ ions while importing only two K^+^ ions. Insulin promotes translocation of Na^+^-K^+^ ATPase from an intracellular pool to the cell membrane. Insulin induces phosphorylation of FXYD1 (phospholemann) by atypical protein kinase C, which leads to an increase in the V_max_ of Na-K ATPase. Insulin also activates the sodium/ hydrogen exchanger-1 (NHE-1) and hence increases the electro-neutral entry of Na^+^ into cells. [26] Using hyperinsulinemic euglycemic clamp technique, Nguyen et al.,[27] have shown insulin-stimulated intracellular uptake of glucose and potassium are independent of each other, and hence the reduced glucose uptake in skeletal muscle, characteristic of insulin resistance in type II diabetes is not associated with a decrease in uptake of K^+^. [28]

To date, there have been three systematic reviews which have evaluated the role of insulin in the treatment of hyperkalemia, all of which have inherent limitations.[4, 29, 30] The first of these studies, by Mahoney et al., only included randomized control trials, and therefore is missing a lot of ‘real-world’ data. In contrast, our study had broad inclusion criteria to capture all the relevant literature, and included patients at high risk for the development of hyperkalemia. The studies by Ahee and Crowe[29], and by Elliot et al.[4] were more broad in their inclusion criteria than that by Mahoney and colleagues[30]; however in contrast to our study, they did not include a quality assessment thereby limiting its generalizability. Moreover, our review also includes studies published after the other reviews, and therefore builds upon the current literature.

Although most medical resources recommend administering 10 units of short-acting insulin as an intravenous bolus for the management of acute hyperkalemia, our review demonstrated only five reports that were small in size (range 5–12 patients), which examined the effect of the administration of 10 units of insulin on serum K^+^ concentration [12, 16, 22–24] Using 10 units of insulin as a bolus, Allon et al.[12] demonstrated a maximal reduction in serum K^+^ concentration at 45 minutes with a subsequent rise in serum potassium concentration at 60 minutes, such that the mean decrease in serum K^+^ at 60 minutes was only 0.53±0.25. A similar trend was seen in the study by Ljutic et al.[23] where the maximal reduction in serum K^+^ concentration was at 45 minutes and subsequently increased. In contrast, Lens et al.[22], Mushtaq et al.[16, and Chothia et al.[24] demonstrated a maximal reduction at 60 minutes of 1.0±0.1, 0.8±0.25, and 0.83±0.17 mmol/l respectively. Of note, a large dose of glucose was administered in the study by Lens et al (40 grams),[22] and in the study by Chothia et al.[24] (50 grams). Such a large dose of glucose may have led to higher plasma insulin concentrations due to endogenous insulin release. The administration of a larger dose of glucose to cause the endogenous release of insulin and avoid the administration of large dose of exogenous insulin however, cannot be recommended as a strategy to lower serum K^+^ in patients with emergency hyperkalemia especially in critically ill patients, as the high levels of plasma insulin that are required to achieve maximal shift of K^+^ into cells may not be achieved.

Two (Mahajan et al.[17] and Duranay et al.[21]) of three studies[17, 19, 21] where 10–12 units of insulin were administered as an intravenous infusion over 15–30 minutes demonstrated the smallest decrease in serum K^+^ concentrations at 60 minutes (0.3 mmol/L in the study by Duranay et al, and 0.48 mmol/L in the study by Mahajan et al) among all the studies included in our review. Although the study by Ngugi et al.[19] demonstrated the largest decrease in serum K^+^ concentration at 60 minutes (1.14 ±NR mmol/L), it did not report the standard error of the decrease in serum K^+^ concentrations. Of note, this study also reported a significant decrease in serum K^+^ concentrations with bicarbonate therapy alone, which is in contrast to other studies by Allon et al. and by Blumberg et al.[18, 20]

Three studies[15, 20, 25] which infused 20 units of short acting insulin over 60 minutes were included. Of these, the mean fall in serum K^+^ concentration at 60 minutes was appreciably lower in the study by Kim et al.[15] (0.6 ± 0.14 mmol/L) than observed by Allon et al.[25] (0.85±0.06 mmol/L), and by Blumberg et al.[20] (0.92 ± 0.40 mmol/L). It is interesting to note that insulin levels at 60 minutes were much lower in the study by Kim et al.[15] (196±18 uU/ml) than in the study by Allon et al.[25] (492±99 uU/ml), and in the study by Blumberg et al.[20](354±21.79 uU/ml), although a similar dose of insulin was administered.

Data from studies using insulin clamp technique, suggest that an insulin level of 500 iu/ml is required to achieve maximal K^+^ shift (-1.54 mmol /L).[31] The data available with the measurement of plasma insulin levels in studies using an infusion of 20 units of insulin or the administration of 10 units of insulin as an intravenous bolus, suggest that these plasma insulin levels are more likely to be achieved and maintained with the administration of 20 units of insulin as in infusion over 1 hour, rather than with the administration of10 units of insulin as an intravenous bolus. We think it is instructive to examine the two studies by Allon et al.[12, 25] in which two different doses of insulin were given and insulin levels in blood were measured, together with the mean reduction in serum K^+^ concentration.In both of these studies, similar patient populations were enrolled, and similar methodology was employed. In the study, in which insulin was given as a bolus of 10 units, the plasma insulin level at 60 minutes was 61.9±22 uU/ml, and the mean decrease in serum K^+^ was 0.53±0.25 mmol/L. In contrast, in the study in which 20 units of insulin was given as a continuous infusion over 60 minutes, the plasma insulin level at 60 minute was 492±99 uU/ml, and the mean reduction in serum K^+^ concentration was 0.85±0.06 mmol/L. Notwithstanding, the initial serum K^+^ level in the study in which 20 units of insulin were given, was 4.28±0.30 mmol/L, which may have been expected to result in a less fall in serum K^+^, and hence bias the results against the 20 units of insulin regimen.

Hypoglycemia is clearly a major concern when receiving insulin therapy, especially with large doses. In fact, hypoglycemia occurred in about 50% of patients in the study by Blumberg et al.[20] in which 20 units of insulin were given as an infusion over 60 minutes; however, only 25 grams of glucose were infused. The studies by Allon et al.[25] and Kim et al.[15] used a similar dose of insulin; but none of their patients who received an infusion of either 60 grams or 40 grams of glucose had developed hypoglycemia. Of note, plasma insulin levels were lower in the study by Kim et al. Taken together, we would suggest a 60 gram infusion of glucose in patients who are given 20 units of insulin. In studies where 10 units of insulin were infused, the fewest cases of hypoglycemia were seen in patients given 50 grams of glucose. Accordingly, we therefore, suggest the administration of 50 grams of glucose to patients who are given 10 units of insulin. Frequent measurement of plasma glucose levels is mandatory.

Our study has a few limitations. First, there are only a small number of studies with small number of patients included in our review, our search strategy was broad and inclusive, which allowed us to generate the most possible, comprehensive evidence-based review. Second, the studies available in the literature are highly heterogeneous in methodology and patient population, which precluded us from meta-analyzing our results. Third, the limited data in the literature did not allow us to separate patients receiving different insulin regimens, into groups based on whether they had acute kidney injury, chronic kidney disease or end-stage renal disease on hemodialysis. Fourth, in three studies, we extrapolated a weight-based dose (assuming a 70kg male), and this may overestimate the actual dose of insulin given. Fifth, none of the included studies provided detailed information concerning EKG changes associated hyperkalemia, and whether each dosing regimen provided resolution of those changes. However, in a retrospective study of the frequency of EKG changes in patients with hyperkalemia by Montague et al., the EKG had poor sensitivity for diagnosing hyperkalemia, such that only 55% of patients with serum K^+^ > 6.8 mmol/L had peaked T waves. Notwithstanding, 14/90 patients in this case series developed cardiac arrhythmia or cardiac arrest, a number of them did not have any EKG changes on their initial presentation.[32] Sixth, the three studies that used the infusion of 20 units of insulin over 60 minutes, reported data on plasma K^+^ up to only 60 minutes. Although insulin levels were high, we cannot determine if rebound hyperkalemia did occur. Seventh, there was a large variability in the initial serum K^+^ concentration between studies, which may have affected the degree of fall in serum K^+^ concentration in response to the administration of insulin. Finally, hypoglycemia was not explicitly defined in terms of a glucose value in a majority of the included studies, despite being mentioned in the results of these studies. This may have led to heterogeneity in the incidence of hypoglycemia in the included studies.

## Conclusion

Although there are multiple interventions that are used in combination to treat hyperkalemia, insulin is the mainstay of therapy in an emergency setting of hyperkalemia, where a management strategy that can quickly, and reliably lower serum K^+^ concentration is required. The limitations of other currently available interventions to induce a shift of K^+^ into cells or its loss via the GI tract has been previously reviewed.[2, 33, 34] The limited data available in the literature shows no statistically significant difference between the different regimens of insulin used to acutely lower serum K+ concentration. Accordingly, 10 units of short acting insulin given intravenously may be used in cases of hyperkalemia. Alternatively, 20 units of short acting insulin may be given as a continuous intravenous infusion over 60 minutes in patients with severe hyperkalemia (i.e., serum K+ concentration > 6.5 mmol/L) and those with marked EKG changes related to hyperkalemia (e.g., prolonged PR interval, wide QRS complex) as an alternative to 10 units of short acting insulin. Because the risk of hypoglycemia is increased with using large insulin doses, sufficient glucose (60 grams with the administration of 20 units of insulin and 50 grams with the administration of 10 units) should be given to prevent hypoglycemia, and plasma glucose should be frequently monitored. Ultimately, only a high-quality randomized control trial evaluating differences in the efficacy and safety among various regimens of insulin administration in patients with hyperkalemia can definitively answer the question as to which regimen is best.

## Supporting Information

S1 Appendix PRISMA Checklist. (DOC)

S2 Appendix Search Strategy. (DOCX)
