# Supplementary material for: Optimal Dose and Method of Administration of Intravenous Insulin in the Management of Emergency Hyperkalemia: A Systematic Review
Source: PLoS One. 2016 May 5;11(5):e0154963. doi: 10.1371/journal.pone.0154963 (PMC4857926; doi:10.1371/journal.pone.0154963)
Supplement: S2 Appendix — (DOCX) [file pone.0154963.s002.docx]

**Appendix 2: Search strategy**

**Database: Ovid MEDLINE(R) In-Process & Other Non-Indexed Citations and Ovid MEDLINE(R) <1946 to Present>**

Search Strategy:

--------------------------------------------------------------------------------

1 Hyperkalemia/ (4684)

2 hyperkal*.mp. (8210)

3 hyperpotass*.mp. (182)

4 1 or 2 or 3 (8325)

5 exp Insulin/ (153218)

6 insulin.mp. (304964)

7 5 or 6 (304964)

8 4 and 7 (460)

**Database: EBM Reviews - Cochrane Central Register of Controlled Trials <February 2015>**

**Search Strategy:**

--------------------------------------------------------------------------------

1 Hyperkalemia/ (88)

2 hyperkal*.tw. (285)

3 hyperpotass*.tw. (1)

4 1 or 2 or 3 (311)

5 exp Insulin/ (7666)

6 insulin.tw. (15087)

7 5 or 6 (16041)

8 4 and 7 (30)

**Embase Classic+Embase 1947 to 2015 Week 10**

**1 exp hyperkalemia/ 15929**

**2 hyperkal*.mp. 18952**

**3 hyperpotass*.mp. 551**

**4 1 or 2 or 3 19096**

**5 exp insulin/ 278788**

**6 insulin.mp. 582578**

**7 5 or 6 582578**

**8 4 and 7 2359**
